# Effective Chemical Fixation of CO_2_ into Phenol Using Conventional Gas‐Solid and Novel Suspension‐Based Kolbe‐Schmitt Reactions

**DOI:** 10.1002/cssc.202402564

**Published:** 2025-03-17

**Authors:** Omar Mohammad, Jude A. Onwudili, Qingchun Yuan, Robert Evans

**Affiliations:** ^1^ Energy and Bioproducts Research Institute Aston University, Aston Street Birmingham B4 7ET, West Midlands United Kingdom; ^2^ Department of Chemical Engineering and Biotechnologies Aston University, Aston Street Birmingham B4 7ET, West Midlands United Kingdom

**Keywords:** CO_2_ utilisation, Kolbe-Schmitt reaction, Suspension-based phenol carboxylation, Conventional dry phenol carboxylation, Reaction mechanisms

## Abstract

The Kolbe‐Schmitt reaction is a classic route for CO_2_ utilisation through organic synthesis of industrially relevant chemicals. Despite the reaction's long‐standing history, detailed product separation and analysis remain underexplored, which inherently limits an accurate mechanism elucidation. This study introduces a new comprehensive approach for isolating and characterising reaction products using high‐performance liquid chromatography (HPLC) and proton nuclear magnetic resonance (^1^H‐NMR). Comparative experiments at 225 °C and 30 bar CO₂ were carried out using the conventional gas‐solid and novel suspension‐based methods with varying reaction times. A new two‐step reaction mechanism is proposed. In the first step, 2‐disodium salicylate and phenol are formed. In the second step, 2‐monosodium salicylate is formed, with subsequent regeneration of sodium phenoxide. This mechanism was validated by adding pure (free) phenol to the reaction media in both conventional and suspension‐based methods. The presence of added phenol was found to increase the yield of salicylic acid by 25.0 % and 8.5 % after 2 hours of reaction, for each method, respectively, compared to experiments without added phenol. Successful application of these enhanced carboxylation methods to other biomass‐derived single‐ring phenolic compounds will offer new routes for potential large‐scale CO₂ utilisation.

## Graphical abstract

This study compares conventional gas‐solid and novel suspension‐based Kolbe‐Schmitt reactions in toluene. Using a new methodology for precise product separation and analysis via high‐performance liquid chromatography (HPLC) and proton nuclear magnetic resonance (^1^H‐NMR), we validated a new mechanism through phenol addition, achieving the highest reported salicylic acid yield in 2 hours. These findings advance CO₂‐based carbon capture and utilisation (CCU) pathways.

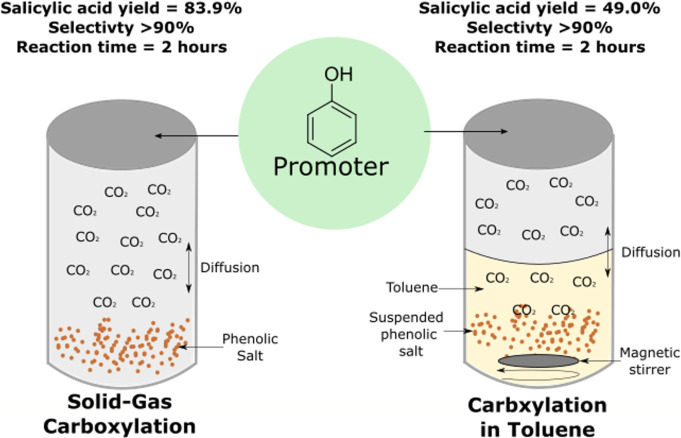

## Introduction

Reaction mechanisms are important for controlling and predicting the course of reactions, thus helping to understand what products can form or how specific reaction steps can lead to the formation of a given product or set of products. They are important for the development of chemical process by modifying reaction pathways to increase the yields of main products and minimise the formation of undesirable side‐products. The link between reaction mechanisms and the scaling up of chemical processes lies in the need for a detailed understanding of how reactions proceed at the molecular level. This is critical when translating a laboratory‐scale reaction to industrial production. Scaling up involves addressing challenges such as heat and mass transfer, mixing efficiency, and maintaining reaction kinetics.^[1]^ Without a clear understanding of the reaction mechanism, it is difficult to predict how these factors will influence product yields, selectivity, and the formation of by‐products at larger scales. Understanding the mechanism also allows engineers to implement process intensification strategies,^[2]^ such as improved mixing, using solvents to enhance selectivity and reducing energy consumption, all in line with the green chemistry principles.^[3]^

Carboxylation reactions of phenols were first reported by Kolbe in 1860^[4]^ and later, with modified reaction conditions, by Schmitt in 1886.^[5]^ The process begins with the preparation of sodium phenoxide (PhONa) by reacting an equimolar amount of phenol with NaOH. In the next step, the dried PhONa solids are reacted with CO_2_ to produce mainly 2‐hydroxybenzoic acid, otherwise known as salicylic acid (SA). By incorporating CO_2_ into aromatic phenol compounds, the reaction has attracted research interest, such as in converting biomass into value‐added platform aromatic acids.[^6^, ^7^] A process that is highly selective, yields a high amount of the desired products, and can withstand continuous processing for high‐throughput manufacturing is needed. The Kolbe‐Schmitt reaction has reported a SA molar yield of 79.0 % after 8 hours at 125 °C and CO_2_ pressures ranging from 82 to 138 bar.^[8]^ The yield is the highest reported in the open literature, however, the rection lacks both a full characterisation of product distribution and a clear mechanism. In addition, the gas‐solid reaction requires an extended reaction time, high CO_2_ pressures, and exhibits inadequate mass and heat transfer when performed in a batch reactor. These factors lead to low productivity, local hotspots and the formation of undesired products. Despite these drawbacks and limitations, the conventional Kolbe‐Schmitt reaction remains in use.^[4]^

Recent research efforts have focused on decoupling mixing from fluid velocity and pressure by suspending PhONa solids in various solvents. A range of solvents, including alcohols (methanol, ethanol, and 1‐butanol), glycol, glycerol, xylene, di‐isobutyl ketone, and more recently, toluene, have been used to modify the traditional Kolbe–Schmitt reaction.[^4^, ^9^, ^10^] Experimental results demonstrated that solvents of low dielectric constants favoured increases in the yield of SA. However, it is important to note that these works lacked full characterisation of product distribution, hence, a clear mechanistic study has still yet to be illustrated.[^4^, ^10^]

**Scheme 1:**
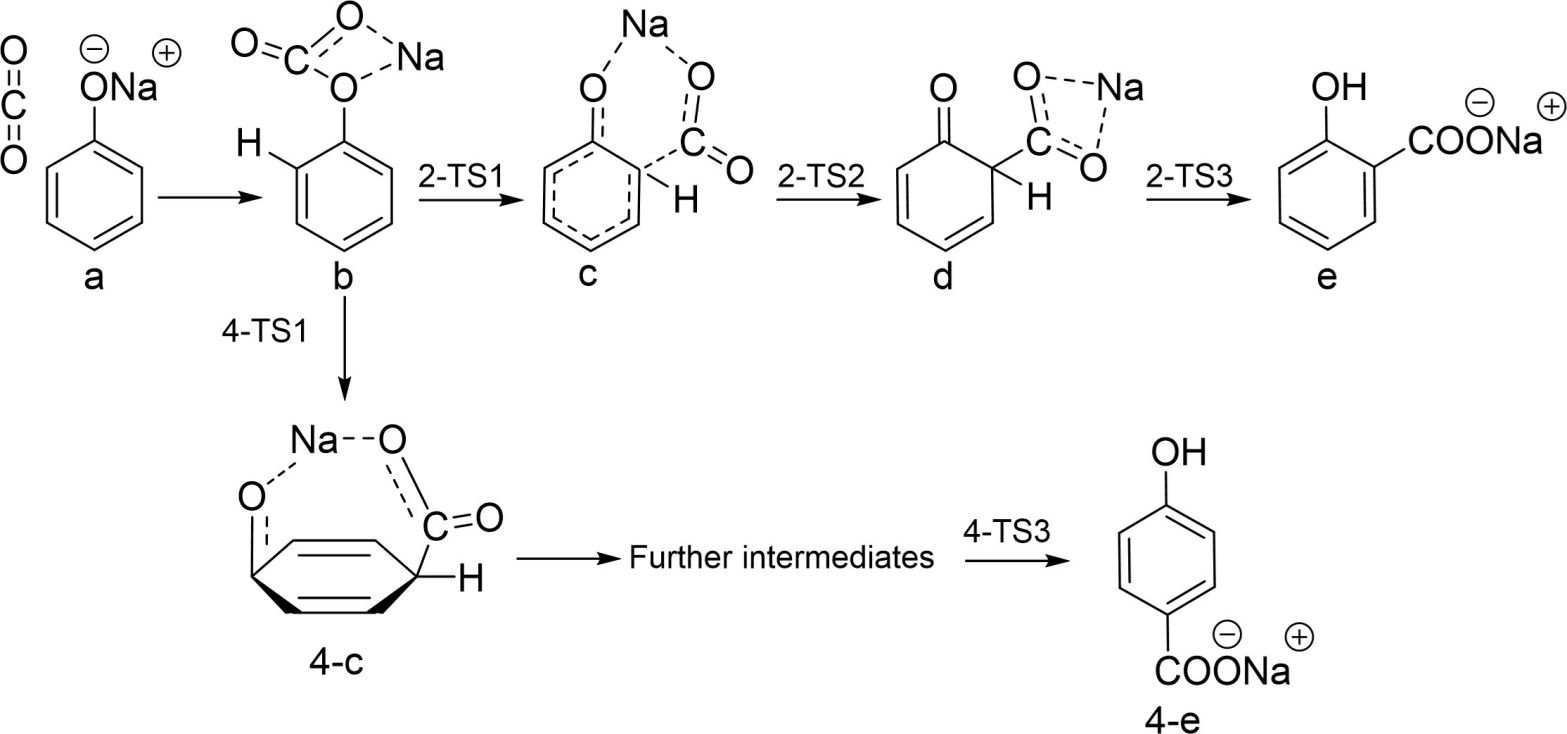
The Kolbe‐Schmitt reaction mechanism proposed by Marković^[13]^, illustrating the formation of intermediates b, c, and d, proceeding through transition states TS1 to TS3.

Earlier studies proposed that PhONa carboxylates to form ′phenyl sodium carbonic acid’ as an intermediate. Schmitt,^[5]^ however, showed that CO₂ added to sodium phenolate does not yield pure sodium phenyl carbonate; rather, sodium salicylate and phenol form directly, bypassing this intermediate.^[5]^ Several experimental and theoretical efforts have been conducted to elucidate the mechanisms of the Kolbe‐Schmitt reaction.[^4^, ^5^, ^11^, ^12^, ^13^, ^14^, ^15^] Density functional theory (DFT) was evaluated as a more accurate approach for quantum chemistry calculations. The DFT study of Markovic^[13]^ revealed that carboxylation under gas‐solid reaction conditions proceeded in four sequential steps via the formation of three transitions states and three intermediates, as indicated in Scheme 1. It was widely accepted that direct carboxylation occurs via formation of PhONa‐CO_2_ complex in the first step (Compound b, in Scheme 1).[^11^, ^12^, ^13^] The fourth step (TS3) is irreversible. However, in the presence of a solvent, the fourth step becomes reversible,^[16]^ leading to low yields of SA. Recent experimental work conducted within our research group supported the idea of reversibility at this stage in the reaction. It was proposed that, in the presence of a solvent, formation of di‐sodium salicylate was favoured, as for every one mole of PhONa, almost one mole of SA and one mole of phenol was formed^[10]^. This observation was also in line with Kolbe's initial theory when the experiment was conducted in an open‐system,^[4]^ however, under the modified conditions by Schmitt, the prevention of phenol volatilisation lead to higher yields of SA.^[5]^

Most studies of the Kolbe‐Schmitt reaction report the yields of the carboxylated products formed during the reaction without including quantitative yields of reaction phenols over time.[^4^, ^17^, ^18^, ^19^] Most of the proposed mechanisms are based on theoretical study, including the effect of solvents.[^11^, ^13^, ^16^] These theories do not always match experimental data; for example, a kinetic model predicted no product formation even with extended residence time in solvent,^[16]^ while recent work indicated that SA yield increased linearly with reaction time.^[10]^ In addition, these theoretical studies failed to propose a mechanism for the formation of other diacid by‐products such as 2‐hydroxyisopthalic acid (2‐HIPA) and 4‐hydroxyisopthalic acid (4‐HIPA), focusing mainly on formation of SA and 4‐hydroxybenzoic acid (4‐HBA). Moreover, most literature described quenching the final reaction mixture with water, followed by acidification and characterisation of the resulting solution. However, under these conditions, the free phenol produced during the reaction would be mixed with water‐soluble products, and upon acidification, unreacted PhONa would convert into phenol.[^4^, ^12^, ^18^, ^20^] As a result, distinguishing between the free phenol formed during the reaction and the unreacted phenol is impossible, making conversion calculations inherently inaccurate.

To address this issue, for the first time, the formation of SA, along with any by‐products and any free phenol produced, have been critically analysed throughout the reaction duration. This was achieved through developing a new method to separate the free phenol from the reaction mixture before product characterisation. This allows the unreacted PhONa to be identified and quantified separately, ensuring that conversion and product distribution can be accurately reported. Consequently, the results obtained have been used to propose a new Kolbe‐Schmitt reaction mechanism that explains the formation of both SA and other side products. To validate the proposed mechanism, the influence of phenol as a promoter was investigated by adding increasing weight percentage to the reaction medium in both the conventional and suspension‐based carboxylation methods. The results of this present work can provide the basis for further research on the understanding of the chemistries of carboxylation of other phenolics. Better understanding of the solvent‐based system will help to develop an industrial scale semi‐continuous synthesis of monohydric phenolic compounds.

## Results and Discussion

### Improved Methodology and Characterisation of Product Distribution Over Reaction Time for the Kolbe‐Schmitt Reaction

**Figure 1:**
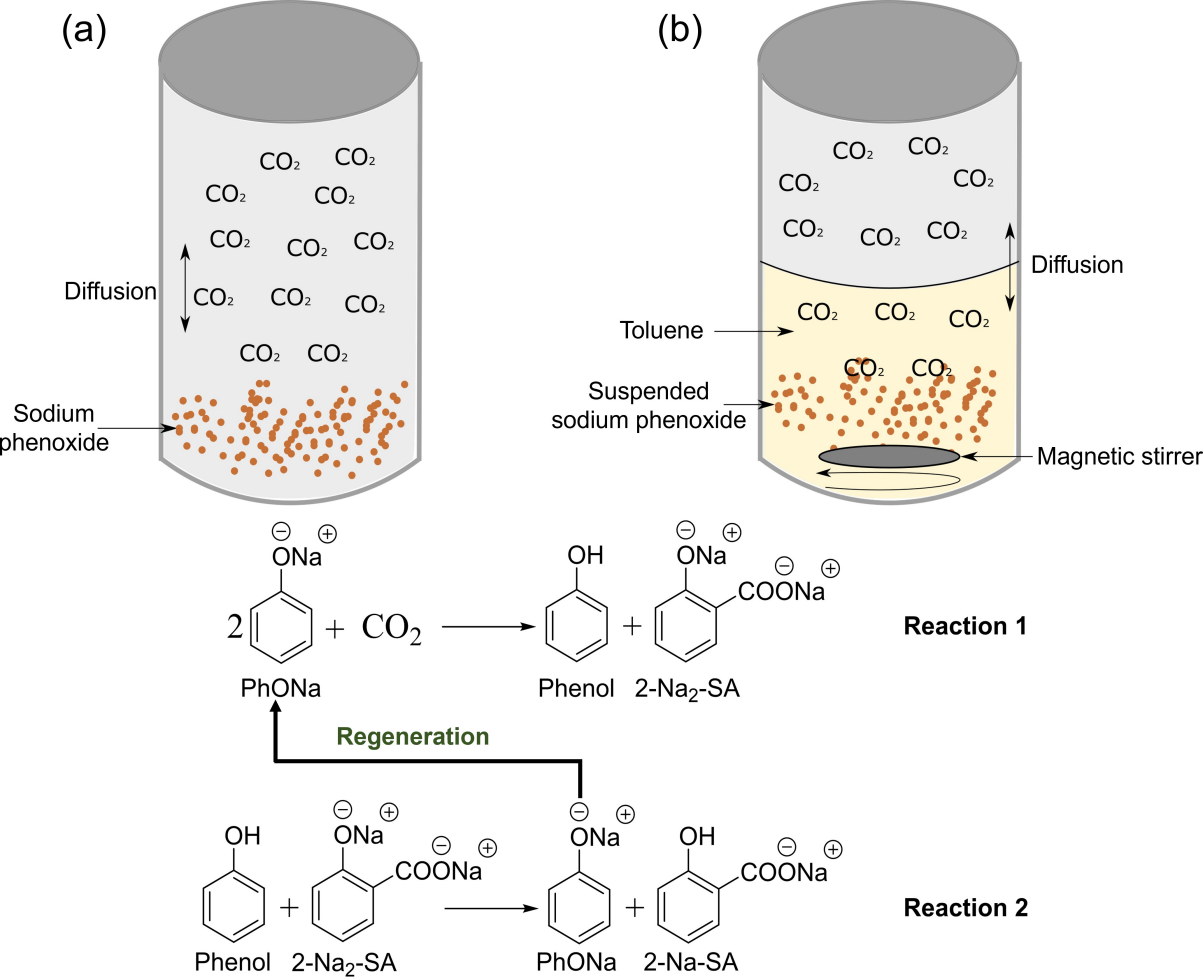
Illustration of the reaction setup for (a) the conventional Kolbe‐Schmitt reaction and (b) the suspension‐based Kolbe‐Schmitt reaction using toluene as an organic carrier. Reaction 1 depicts the first stage leading to the formation of 2‐disodium salicylate (2‐Na₂‐SA) and phenol in the first step, followed by 2‐monosodium salicylate (2‐Na‐SA) and regeneration of sodium phenoxide (PhONa) in the second stage in Reaction 2.

A schematic representation of both reactions is shown in Figure 1, including the main reaction schemes of the Kolbe‐Schmitt reactions. The detailed information of the reactor set‐up and reaction conditions can be found in Supporting Information (Figure S1). One of the key differences in this study compared to others is the development of a methodology to separate the free phenol formed during the reaction from the salts of SA and characterisation of the by‐products. Typically, the reaction products are quenched with water,[^4^, ^12^, ^18^, ^20^] the free phenol formed during the reaction remains with the water‐soluble products. Upon acidification and characterisation of the solution, the free phenol is then assumed to be unconverted PhONa. As for the solid products, in industrial processes, SA is precipitated, leaving behind 4‐HBA in the mother liquor, along with about 15 % of SA, as it is slightly soluble in water. To achieve higher purity, the technical product is sublimated, leaving behind 2‐HIPA and 4‐HIPA.^[21]^ In this present study, the free phenols formed during the reaction were separated with toluene first. In the next step, concentrated HCl (12 M) was added dropwise to the salts of hydroxybenzoic acids (HBAs) and then dried to obtain the organic form of SA and its by‐products. This approach ensured no products were lost in the mother liquor, allowing for a detailed mass balance to explain the formation of SA and its by‐products from the experiments. The experimental set‐up and methodology are depicted as an infographic in Figure S2. After the reaction, several gas samples were analysed by a gas chromatograph according to previously reported methods^[10]^. The chromatograms indicated no detection of hydrocarbon gases (Figure S3 (a)), with only unreacted CO₂ present (Figure S3 (b)).

Experiments were conducted at 225 °C, well below the 240 °C thermal decomposition temperature of salicylic acid.[^10^, ^22^] No char formation was observed. Charring of the reaction mixture at 210 °C has been reported in literature when PhONa was reacted in the presence of 18.75 % moisture content,^[23]^ different conditions from those used in this study. The presence of water significantly affects the reaction, as it has been reported that water hydrolyses PhONa into water and phenol.[^4^, ^10^, ^24^] To minimise moisture, the prepared PhONa was carefully handled prior to the reaction (see Supporting Information). The purity of PhONa was assessed using two methods via acidification of PhONa (Scheme S1): (1) back‐acidification and NaCl recovery, which resulted in 100 % recovery of NaCl (Table S1), and (2) acidification of PhONa followed by phenolic quantification via high‐performance liquid chromatography using external standard method (Figure S4), with phenol recovery determined to be 97.6 % ±0.70 %. These results confirm the high purity of the PhONa, ensuring accurate mass balance for subsequent reactions.

**Figure 2:**
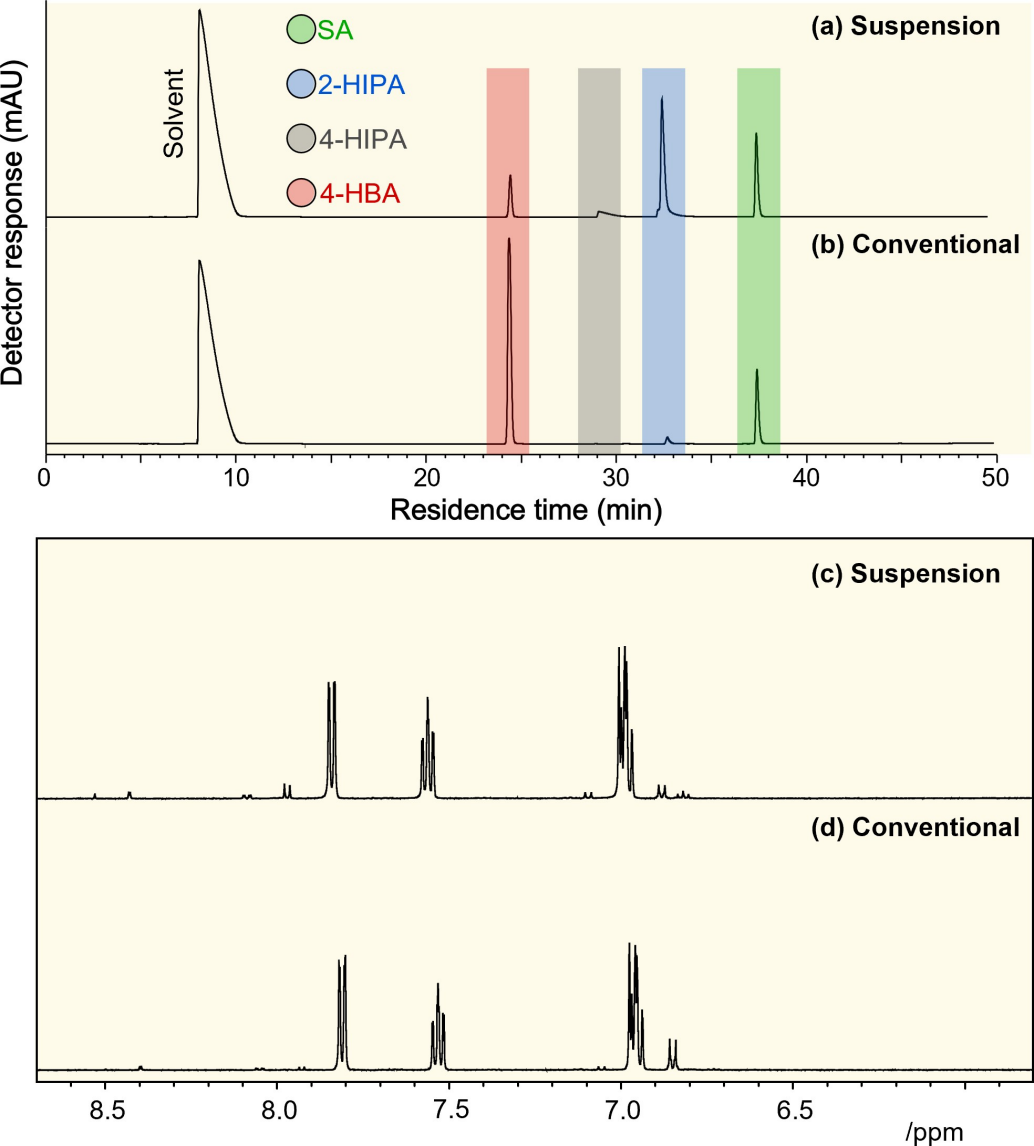
HPLC and NMR spectra comparing the Kolbe‐Schmitt reaction products under (a, c) suspension‐based and (b, d) conventional processes after 2 hours of reaction. HPLC analysis reveals a larger peak area for 2‐hydroxyisophthalic acid (2‐HIPA) and minor formation of 4‐hydroxyisopthalic acid (4‐HIPA)in the suspension‐based process, while the conventional process shows a higher peak area for 4‐HBA with no detection of 4‐HIPA. The NMR spectra further confirm these differences, with both methods showing dominant signals for salicylic acid (SA), the primary product (~90 %). A full description of the NMR multiples and their analysis can be found in Supporting Information.

The free phenol produced from carboxylation of sodium phenoxide was extracted with toluene from the reactor and quantified using gas chromatography/flame ionisation detector (GC/FID) and gas chromatography/mass spectroscopy (GC‐/MS) (Figure S5). The solid products were further processed to obtain SA (Figure S2. A new method using high‐performance liquid chromatography system integrated with UV detector (HPLC‐UV) was developed for enhanced separation and detection of SA and other by‐products (Figure 2 (a and b)). Notably, 4‐HBA exhibits stronger absorption at 254 nm compared to SA. Therefore, the larger peak observed for 4‐HBA in Figure 2 (b) is not indicative of a higher quantity but rather a result of its greater absorption at this wavelength. The product composition (wt %) derived from HPLC data, used to calculate molar yields based on a 1 : 1 molar ratio of PhONa to HBAs as shown in Scheme S2, is summarised in Tables S2–S5. Nuclear magnetic resonance (NMR) spectroscopy, particularly pulsed field gradient (PFG) NMR and diffusion ordered spectroscopy (DOSY),[^25^, ^26^] was also used to analyse the product mixtures. The chemical shift regions between 6 and 8.5 ppm were used to successfully identify the aromatic protons (Figure 2 (c and d)). The NMR analysis provided additional quantitative information to support the accuracy of the HPLC analysis of the conventional and suspension‐based Kolbe‐Schmitt reactions. The major product identified by the NMR was clearly SA, which exhibited four NMR peaks, 2 doublets and 2 triplets. The para‐product (4‐HBA) showed a pair of doublets. The other minor products, 2‐HIPA and 4‐HIPA, could be identified using DOSY spectra as the peaks corresponding to these larger species aligned with a lower diffusion coefficient, ~2.2–2.3×10^−10^ m^2^ s^−1^ (Figure S6). The presence of 2‐HIPA and 4‐HIPA in the product mixture aligns with previous reports in the literature and in a patent.[^4^, ^21^]

In terms of quantitative NMR analysis, each single peak corresponds to a single proton environment. By allocating at least one peak observed to one of the four possible products (Figure S7), integrals were measured and consequently, the purity of each of the compounds could be estimated (Figure S8). The quantitative NMR analysis gave ± 2 % difference to the HPLC method highlighting its accuracy (Table S6).

The effect of time on the yield and composition of SA produced at the previously obtained conditions was tested^[10]^. In the suspension‐based process, after one hour of reaction time, the formation of phenol was favoured, with a total molar yield of 51.2 % and a SA yield of 37.0 %, achieving 89.9 % conversion. In contrast, the conventional process yielded almost equimolar amounts of HBAs (45.0 %) and phenol (45.7 %), respectively, supporting the main reaction equation, **Reaction 1**, in Figure 1. Hence, these results indicate high conversion (90.8 %) of PhONa. The first hour of the reaction proved crucial, as a significant amount of phenol was produced then. As the reaction time increased, for both the conventional and suspension‐based processes, the molar yield of phenol decreased, with a corresponding increase in the formation of HBAs, particularly SA. For instance, the maximum yield of SA in the suspension‐based reaction occurred at 8 hours, with a molar yield of 47.0 %, and a total HBAs yield of 55.0 % while the phenol yield was 42.9 %. In the conventional process, the highest SA molar yield (63.9 %) was reached after 6 hours, with a phenol yield of 21.0 %. Notably, at 8 hours of reaction time, the SA yield dropped significantly to 38.0 %, without affecting the yields of other HBA side‐products. This decline in yield has been previously attributed to a secondary reaction involving the conversion of 2‐Na‐SA, which, in the absence of phenol, reacts with itself to form one mole each of 2‐Na_2_‐SA, phenol, and CO₂.^[10]^

### Proposed Mechanism for Kolbe‐Schmitt Reaction Products Based on Experimental Results

**Scheme 2:**
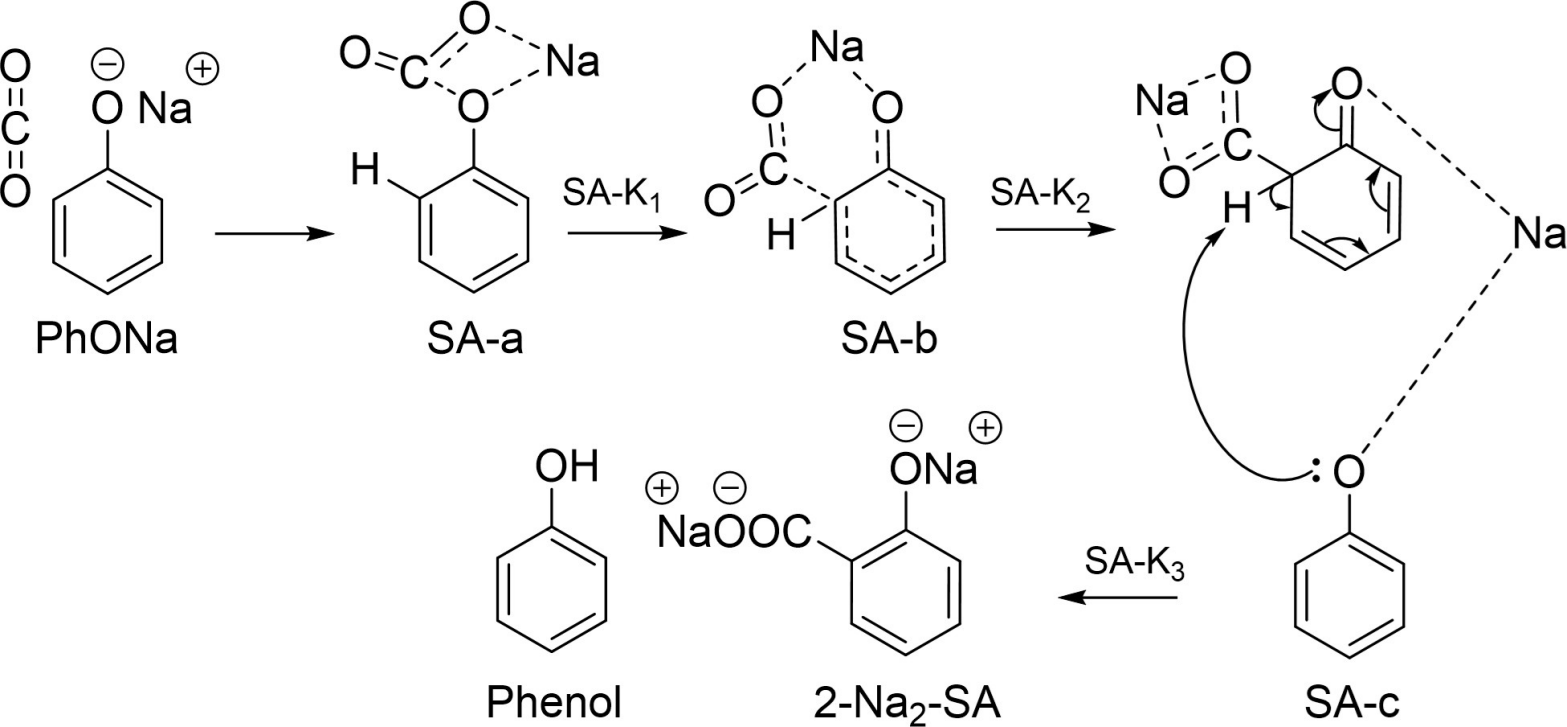
Proposed mechanism based on experimental results for the Kolbe‐Schmitt reaction. The final stage involves proton‐sodium substitution through between the intermediate and PhONa to form SA‐c, yielding one mole each of disodium salicylate (2‐Na_2_‐SA) and phenol.

Previous reports widely accepted that direct carboxylation occurs via formation of PhONa‐CO_2_ complex in the first step (Compound b in Scheme 1),[^11^, ^12^, ^13^] which is followed by deprotonation of the carbon atom and then the protonation of the oxygen atom through the transition state TS3. However, this final step is reversible in the presence of a solvent.^[16]^ Markovic^[13]^ mentioned that this step requires significant amount of energy, and does not justify the formation of phenols. Hence, based on the experimental results, the final stage is believed to involve proton‐sodium substitution between the intermediate and PhONa to form one mole of each 2‐Na_2_‐Sal and phenol. As the reaction proceeds, 2‐Na_2_‐Sal reacts with phenol to form 2‐Na‐Sal and regenerate PhONa which undergoes further successive reactions (Scheme 2).

**Figure 3:**
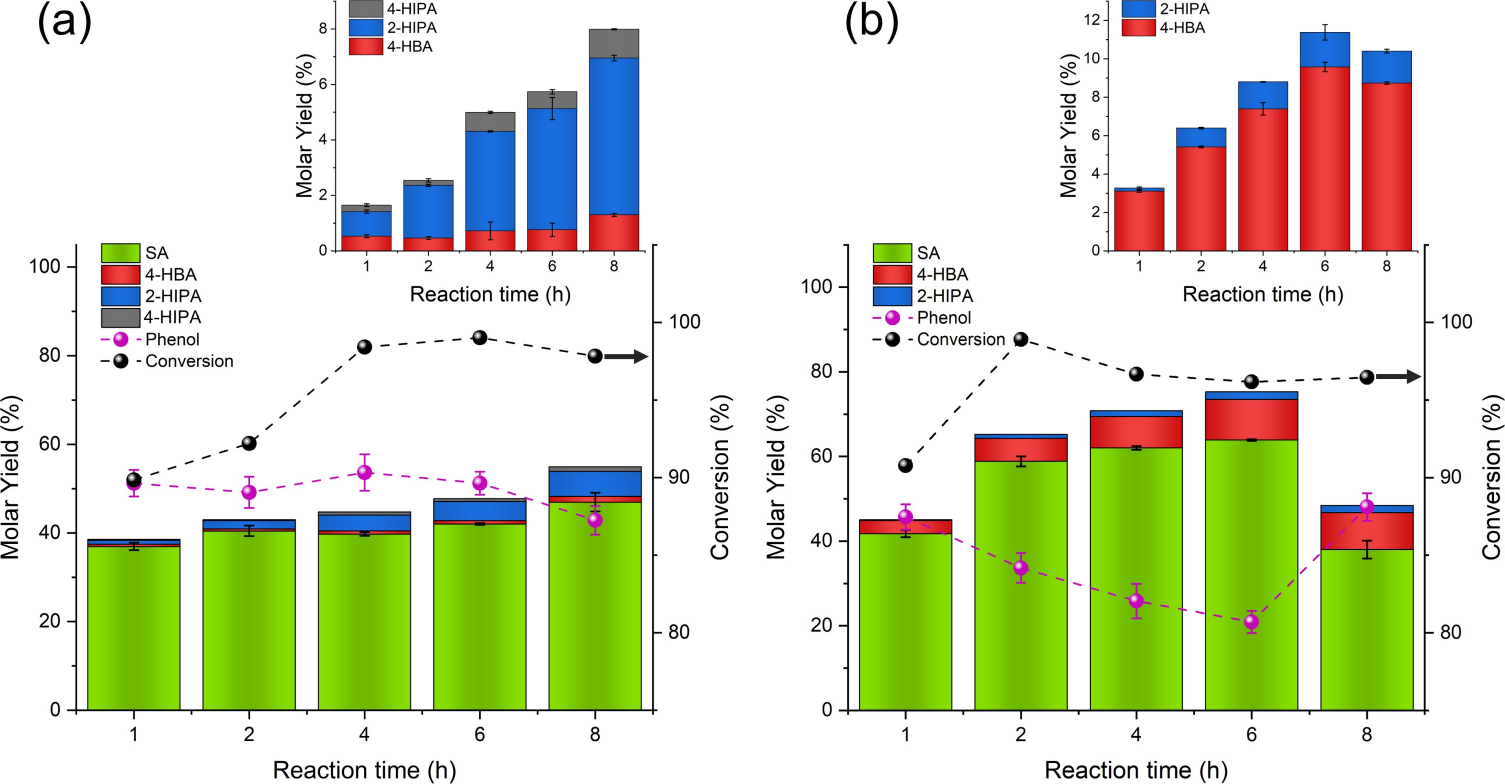
Molar yields of SA, 4‐HBA, 2‐HIPA, −4‐HIPA, and phenol, along with conversion, for the Kolbe‐Schmitt reaction under (a) suspension‐based and (b) conventional processes. The suspension process shows higher yields of 2‐HIPA and minor 4‐HIPA formation, while the conventional process favours 4‐HBA. SA is the dominant product in both cases, with conversion peaking >90 % earlier in the reaction.

Figure 3 indicates that the formation of 4‐HBA is favoured in the conventional process, whereas 2‐HIPA is favoured in the presence of solvent, with minor amounts of 4‐HIPA also detected. In the suspension‐based reaction, the yield of 4‐HBA remained consistently low, with a slight increase from 0.53 % at 1 hour to 1.30 % after 8 hours. This modest rise suggested that the solvent likely inhibited para‐carboxylation, possibly due to solvation effects or steric hindrance around the phenoxide ion, reducing the accessibility of the para position for CO_2_ addition.[^27^, ^28^] Conversely, in the conventional process, the yield of 4‐HBA increased significantly, from 3.11 % at 1 hour to 9.57 % after 6 hours, although it slightly decreased after this point as overall yields began to decline. The sustained increase in 4‐HBA yield in the conventional process indicates that, without a solvent present, CO₂ has better access to the para position on PhONa, promoting para‐carboxylation over time.

The formation of di‐carboxylated HBAs such as 2‐HIPA and 4‐HIPA could be interlinked with the formation of 2‐Na_2_‐SA and phenol in the first step. In the suspension‐based reaction, the interaction between phenol and 2‐Na_2_‐SA (Figure 1, Reaction 1) proceeds relatively slowly. This slower rate makes the formation of di‐carboxylated products like 2‐HIPA competitive with the formation of SA. Observations show that the yield of 2‐HIPA begins at 0.89 % and increases linearly over time, reaching 5.65 % after 8 hours.

In contrast, it is plausible to consider that the rate of **Reaction 1** in Figure 1 appears to be intrinsically faster in the conventional process, favouring the rapid formation of 2‐Na‐SA and PhONa from 2‐Na₂‐SA and phenol. This could be a limitation for di‐carboxylation, as 2‐Na_2_‐SA is quickly consumed before it can react with a second CO₂ molecule. Consequently, the yield of 2‐HIPA remained at 1.66 %, even after 8 hours of reaction time.

**Scheme 3:**
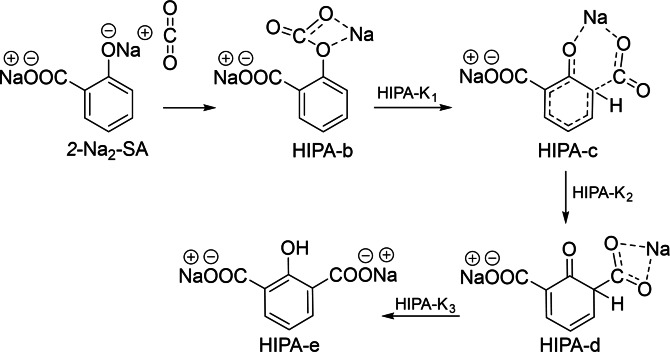
Proposed mechanism for the formation of 2‐hydroxyisophthalic acid (2‐HIPA) in the presence of disodium salicylate (2‐Na_2_‐SA). The electron‐withdrawing effects of phenoxide (O^−^) and carboxylate (COO^−^) enhance ortho nucleophilicity, promoting a second CO₂ attack. The pathway involves three intermediates (HIPA‐b, HIPA‐c and HIPA‐d) and three transition states (HIPA‐K_1_, HIPA‐K_2_, HIPA‐K_3_) as supported by Marković’s theoretical study.^[13]^

Mechanistically, the formations of 2‐HIPA and 4‐HIPA are favoured when disodium salicylate is present. According to Hammett's Equation,^[29]^ the presence of both O^−^ (phenoxide) and COO^−^ (carboxylate) groups in 2‐Na₂‐SA exerts a strong electron‐withdrawing effect on the aromatic ring. While this creates an electron‐deficient aromatic system, the phenoxide resonance ensures that nucleophilicity is maintained predominantly at the ortho position. Such a situation is liable to make the ring more reactive toward a second CO₂ attack, leading to the formation of di‐carboxylated products. Based on this mechanism and supported by the theoretical study of Marković,^[11]^ a pathway for 2‐HIPA formation is proposed in Scheme 3. In contrast, monosodium salicylate, with single carboxylate and hydroxyl groups, experiences less electron withdrawal, leaving the ring less nucleophilic at the ortho position, which becomes less prone to a second ortho‐carboxylation step. As a result, monosodium salicylate predominantly forms SA rather than undergoing di‐carboxylation. 4‐HIPA is thought to follow a similar route, considering that its yields are low due to the already limited formation of 4‐HBA.

The pattern observed also aligns with March's mechanistic framework,^[30]^ where 2‐Na_2_‐SA has the required electronic structure for di‐carboxylation, while monosodium salicylate, being less electron‐deficient, cannot support the second carboxylation. This observation is consistent with Lindsey's review,^[4]^ which highlights that di‐metalated derivatives of alkali metal phenoxides can yield SA, 2‐HIPA, and 4‐HIPA as potential products. The proposed mechanism in Scheme 3 (based on reaction steps in Figure 1) is further supported by experimental data: when phenol was added to both the suspension‐based and conventional reactions, confirming that 2‐Na_2_‐SA formation is a critical intermediate in driving mono‐carboxylation and preventing di‐carboxylation pathway as described below.

Effect of Phenol Addition and Validation of the Proposed Mechanism

To validate the proposed mechanism in Scheme 3, a known mass of phenol was added to the reaction to assess its influence on the yield of SA. Based on the mechanism, phenol plays a critical role in facilitating the transformation of 2‐Na₂‐SA and the phenol produced during the initial reaction step (**Reaction 1**, Figure 1) into 2‐Na‐SA and regenerating PhONa (**Reaction 2**, Figure 1). The regenerated PhONa then re‐enters the reaction cycle, driving further transformations. Based on the experimental results, it is considered that the addition of phenol significantly increases the formation of 2‐Na‐SA and PhONa from 2‐Na₂‐SA, thereby enhancing the overall yield of SA. The production of non‐salicylic acid by‐products is crucial, as the isolation and separation of these compounds significantly increase production costs.^[21]^ Among the by‐products, 4‐HBA is easily removed during precipitation due to its higher solubility in water compared to SA. However, 2‐HIPA and 4‐HIPA are more difficult to separate during the purification of SA. To achieve higher purity, the technical‐grade product is typically sublimated, leaving behind 2‐HIPA and 4‐HIPA in the residue.[^4^, ^21^] Therefore, the goal is to maximise SA yield while minimising by‐product formation.

It has been observed that extending the reaction time in the Kolbe‐Schmitt process, while increasing overall yield, leads to higher by‐product formation, which is undesirable. Conversely, higher reaction temperatures favour the formation of SA while reducing 4‐HBA production.^[21]^ Based on the proposed mechanism, it could be crucial to conduct the reaction at shorter reaction times and elevated temperatures to suppress 4‐HBA formation. Additionally, it was anticipated that the introduction of phenol could promote the formation of 2‐Na‐SA while regenerating PhONa, offering the dual benefit of increasing SA yield and reducing the formation of di‐carboxylated by‐products such as 2‐HIPA. Therefore, for both the suspension and conventional process, an optimum reaction time of 2 hours was selected, as full conversion was achieved without significant formation of by‐products. Phenol to PhONa in wt % was added in this ratio, 0 %, 20 %, 50 % and 100 %. Further increase in phenol did not improve the overall yield for the suspension process, as it is suspected to have reduced the solubility of CO_2_ in toluene.^[10]^

**Figure 4:**
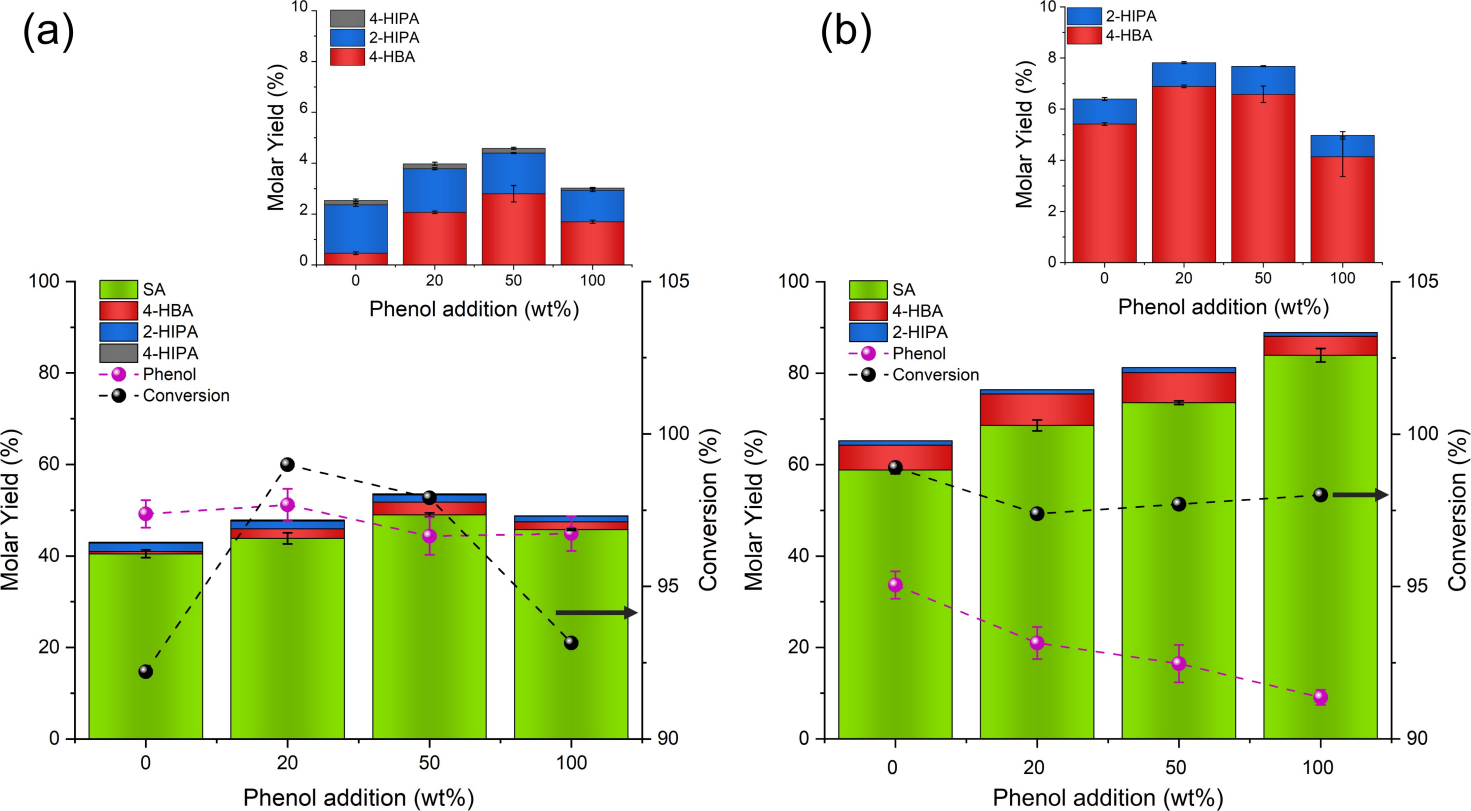
Effect of phenol addition on the Kolbe‐Schmitt reaction under (a) suspension‐based and (b) conventional processes. Increasing phenol concentration results in higher SA yields in both processes, while reducing the formation of 2‐HIPA. The yield of 4‐HBA increased with phenol addition, highlighting the influence of phenol in favouring monosodium salicylate formation and enhancing competition between ortho‐ and para‐carboxylation pathways.

The results in Figure 4(a) of the suspension‐based reaction in toluene show a clear increase in the overall yield of SA with increasing phenol addition up until 50 wt %. As the phenol/PhONa ratio increased from 0 % to 20 % and 50 wt %, the molar yield of SA rose from 40.5 % to 43.9 % and finally to 49.0 %. Correspondingly, the yield of phenol decreased to 49.2 %, 47.0 %, and 44.4 %. The yield of 2‐HIPA also decreased incrementally with the addition of phenol, leading to an increase in the SA/2‐HIPA ratio from 16.2 to 19.4 and 23.2, respectively. A modest decrease in the SA molar yield to 45.8 %, along with corresponding decreases in by‐products, was observed when phenol addition was further increased to 100 wt %. This decrease may be attributed to the overall solubility of CO_2_ in toluene, which could be limited with increase in phenol concentration. CO_2_ can be more soluble in non‐polar solvents,^[31]^ and the presence of excess phenol could alter the overall polarity of toluene.^[32]^ Preliminary tests showed less mass intake of CO_2_ in the reaction mixture with increasing addition of phenol in toluene, the observed differences in CO₂ uptake may be indicative of altered CO₂ solubility in the presence of phenol, see Supporting Information, Table S7.

Overall, these results support the hypothesis that the formation of 2‐Na‐SA is favoured, while di‐carboxylation can only occur in the presence of 2‐Na_2_‐SA. Another notable observation is that the yield of 4‐HBA increased with the addition of phenol, rising from 0.46 % to 2.07 % and 2.80 %, respectively (Figure 4a). The increase in 4‐HBA when phenol addition increased from 20 wt % to 50 wt % can be attributed to the higher concentrations of monosodium salicylate formed in the presence of phenol, which enhances the competition between ortho‐ and para‐carboxylation pathways, thereby promoting the formation of 4‐HBA. In contrast, the yield of 4‐HIPA remained unaffected, staying consistently below 0.20 %

The results presented in Figure 4(b) for the conventional Kolbe‐Schmitt reaction clearly demonstrate the influence of phenol as a promoter on the overall yield of SA when the reaction is conducted in 2 hours. As the proportion of phenol increased from 0 %, 20 %, 50 % to 100 %, the total yield of HBAs rose significantly from 65.3 % to 76.4 %, 81.3 % and 88.9 %, respectively. Such increases highlight phenol's role in enhancing HBA formation by promoting the formation and stabilisation of monosodium salicylate. This favours both ortho‐ and para‐carboxylation pathways, leading to the formation of SA and 4‐HBA. Specifically, the yield of SA increased markedly from 58.9 % to 68.6 %, 73.6 % and finally to 83.9 %, reflecting the increasing preference for monosodium salicylate formation over di‐carboxylated products such as 2‐HIPA, whose yield remained in the range of 0.93 %‐1.10 %. The low yield of these byproducts is consistent with previous findings,[^4^, ^21^] where the rapid formation of 2‐Na‐SA in the conventional process limits the availability of 2‐Na_2_‐SA for a second CO₂ attack. In contrast, the yield of phenol decreased significantly, from 33.7 % to 21.0 %, 16.5 and 9.01 %, indicating that PhONa is being consumed more efficiently as it is regenerated. It is possible that this promotes faster 2‐Na‐SA formation due to the greater accessibility of phenol to react faster with 2‐Na_2_‐SA in the initial stages of the reaction. A slight increase in the yield of 4‐HBA was also observed, rising from 5.42 % to 6.58 %, indicating a minor shift towards para‐carboxylation with increasing phenol content. However, the overall change remained modest, likely due to the overall increase in yield, as the SA/4‐HBA ratio stayed within the range of 9.95 %‐11.2 % up to 50 wt % phenol addition. Interestingly, increasing the phenol addition to 100 wt % not only raised the SA yield to 83.9 %, but also further reduced the formation of 4‐HBA to 4.14 % and 2‐HIPA to 0.83 %

Under similar reaction conditions, the conventional method showed superior performance after 2 hours of reaction compared to the suspension‐based approach, in terms of SA yields. A major challenge is that the current global production capacity of HBAs utilises only around 41,450 tonnes of CO_2_ annually representing a mere 0.00012 % of the 35 billion tonnes of annual global anthropogenic CO_2_ emissions.^[6]^ Therefore, efforts are needed improve the value chain of HBAs e. g., through their large‐scale application to drive up production quantities and hence, overall CO_2_ utilisation. The suspension‐based approach shows promise for large‐scale, continuous carboxylation of phenolic compounds. Moreover, these experimental results provide a foundation for further optimisation, including the exploration of different solvents, solvent/PhONa/phenol ratios, mixing methods and pressures.

## Conclusions

There are still challenges to fully understanding the Kolbe‐Schmitt reaction. For example, accurately quantifying phenols over time remains challenging due to the mixing of phenol produced during the reaction and the one produced from unreacted sodium phenoxide during quenching and acidification. Theoretical models of the reaction often fail to match experimental data or account for complex diacid by‐products like 2‐HIPA and 4‐HIPA, focusing mainly on SA and 4‐HBA formation. This poor elucidation of the reaction mechanism has led to unsustainable production of various HBAs via the conventional gas‐solid Kolbe‐Schmitt reaction. Therefore, we have proposed a new mechanism for the Kolbe‐Schmitt reaction that explains the higher SA yields observed in the conventional process compared to the suspension‐based reaction using toluene as a dispersion medium. Experimental results demonstrated that disodium salicylate forms in the initial stage of the Kolbe‐Schmitt reaction, and then subsequently reacts with phenol in the second stage to produce monosodium salicylate, forming SA or 4‐HBA, while regenerating PhONa for further reaction cycles.

In the conventional process, the highest SA molar yield of 63.9 % was reached at 6 hours, while the suspension‐based process achieved a maximum yield of 47.0 % at 8 hours. Although extended reaction times increased SA yields in both cases, they also led to higher by‐product formation; primarily 4‐HBA for the conventional process and 2‐HIPA for the suspension‐based reaction. Observations of product composition and free phenol formation over time revealed that over 90 % conversion was achieved within just 2 hours for both processes. The proposed mechanism suggests that free phenol plays a critical role in increasing SA yield by accelerating the formation of monosodium salicylate and reducing the production of diacid by‐products. Therefore, conducting the reaction at 2 hours was deemed optimal, as it results in near‐complete conversion with lower by‐product yields.

To validate the proposed mechanism, phenol was added to PhONa at various concentrations. The added phenol was found to increase the yield of SA by 25.0 % and 8.5 % after 2 hours of reaction, for each method, respectively, compared to experiments without added phenol. This discovery is groundbreaking in two ways; (1) it enhanced SA production at short reaction times; and (2) it minimised the formation of by‐products. Although the suspension‐based reaction yielded lower amounts of SA compared to the gas‐solid reaction, it presents the possibility for continuous operation, eliminating the need for long heating and cooling cycles, and extended loading and unloading cycles. These changes would save both time and energy, and hence lead to lower production costs as well as limiting by‐product formation.

## Supporting Information

The authors have cited additional references within the Supporting Information.[^33^, ^34^, ^35^, ^36^]

## Conflict of Interests

The authors declare no conflict of interest.

## Supporting information

As a service to our authors and readers, this journal provides supporting information supplied by the authors. Such materials are peer reviewed and may be re‐organized for online delivery, but are not copy‐edited or typeset. Technical support issues arising from supporting information (other than missing files) should be addressed to the authors.

Supporting Information

## Data Availability

The data that support the findings of this study are available in the supplementary material of this article.
