# Supplementary material for: Effective Chemical Fixation of CO2 into Phenol Using Conventional Gas‐Solid and Novel Suspension‐Based Kolbe‐Schmitt Reactions
Source: ChemSusChem. 2025 Mar 17;18(12):e202402564. doi: 10.1002/cssc.202402564 (PMC12175050; doi:10.1002/cssc.202402564)
Supplement: Supplementary file 1 — Supporting Information [file CSSC-18-e202402564-s001.pdf]

ChemSusChem

Supporting Information

Effective Chemical Fixation of CO_2 into Phenol Using Conventional Gas-Solid and Novel Suspension-Based Kolbe-Schmitt Reactions

Omar Mohammad, Jude A. Onwudili,* Qingchun Yuan, and Robert Evans

WILEY-VCH

Supporting Information

Effective fixation of CO_2 into phenol using improved gas-solid and suspension-based Kolbe-Schmitt reactions

Omar Mohammad[a], Jude A. Onwudili*[am], Qingchun Yuan,[a,b], Robert Evans,[b,c]

[a] O. Mohammad, J.A. Onwudili, Q. Yuan.
Energy and Bioproducts Research Institute,
Aston University, Aston Street, Birmingham, B4 7ET, West Midlands, United Kingdom
Corresponding Author's E-mail: j.onwudili@aston.ac.uk
[b] J.A. Onwudili, Q. Yuan, R. Evans.
Department of Chemical Engineering and Biotechnologies
Aston University, Aston Street, Birmingham, B4 7ET, West Midlands, United Kingdom

WILEY-VCH

Materials

The chemicals that were used for these experiments included phenol (C_6H_5OH, 94.11 g/mol, 99% extra pure; Thermo Scientific™, Waltham, MA USA), sodium hydroxide (NaOH, 39.997 g/mol, ≥98%, Honeywell, Skimped Hill Ln, UK), sodium phenoxide (C_6H_5ONa, 116 g/mol, 98%; Thermo Scientific™), salicylic acid ($C_6H_4(OH)COOH$, 138 g/mol, +99%; Thermo Scientific™),4-hydroxybenzoic acid ($(OH)C_6H_4COOH$, 138 g/mol 99+%,Thermo Scientific™), 2,3-dihydroxyterephthalic acid ($(HO)_2C_6H_2(COOH)_2$,198.13 g/mol, 97%, Thermo Scientific™), de-ionised water (Q-pod system, 0.22 µm, carbon dioxide (CO_2, 44.01 g/mol, CP Grade; BOC), toluene (C_7H_8, 92.13 g/mol, 99.5%, ACS reagent, Thermo Scientific™), and acetone ($(CH_3)_2CO$, 58.08 g/mol, 99.5%, HPLC Grade, Thermo Scientific™).

Preparation of phenolic salt and Analysis using Thermogravimetric Analysis (TGA)

The sodium phenoxide was synthesised according to Kolbe's method. In the initial step, slightly more than one mole of the phenol was dissolved in an equimolar solution of sodium hydroxide (50 wt%). The mixture was loaded in a glass-lined 450 ml 4575A fixed head bench top Parr reactor vessel, equipped with a stirrer. The production of sodium phenoxide was performed at 130 °C for 4 h at a stirring rate 50 rpm. After the reaction, the reactor was cooled down to 40 °C (cooling below 40 °C caused solidification of the salt, presenting difficulty with recovery). The solution containing sodium phenoxide was then transferred into a separate beaker and left inside a vacuum oven at 40°C overnight to dry. The dry sodium phenoxide was removed from the beaker, crushed to particle size of 125 - 250µm and stored in an air-tight container. Before use in reactions, the PhONa was further dried at 100°C under vacuum overnight to eliminate residual moisture and phenol.

The information for the gravimetric analysis (TGA) can be found in a recent publication [1].

Carboxylation reaction – Conventional/Suspension

The conventional and suspension-based carboxylation reactions were conducted in a set of 4 × 10 mL Quadracell reactors **(Figure S1)**, supplied by Asynt (Isleham, Cambridgeshire, United Kingdom). In each reactor cell, a measured amount of sodium phenoxide (0.3 g) was added. For the suspension-based reaction, 6 mL of toluene was mixed with the sodium phenoxide to form a suspension. All four reactor cells were then sealed onto the main reactor cap and purged with CO_2 to remove any residual air.

The reactor system was pressurized to the target operating pressure (30 bar) using a two-stage piston cylinder regulator (GASARC, Tech Master GPT420 Series). The regulator valve controlled the maximum delivery pressure, and a digital pressure transducer monitored the pressure until saturation, indicating that no further CO_2 dissolution in the organic carrier occurred in the suspension-based reactor. The reactor was weighed before and after CO_2 pressurisation to determine the exact mass of CO_2 introduced.

The reactors were heated to the desired temperature (225°C) at a rate of 10 °C/min, with a stirring speed of 1000 rpm for the suspension-based system. The reaction mixture was maintained at the target temperature for 2 hours before being allowed to cool to room temperature. Reaction temperature and stirring speed were controlled using an Asynt ADS-HP-NT magnetic stirrer hotplate, and pressure readings were displayed via a digital pressure gauge integrated with a cooling tower.

WILEY-VCH

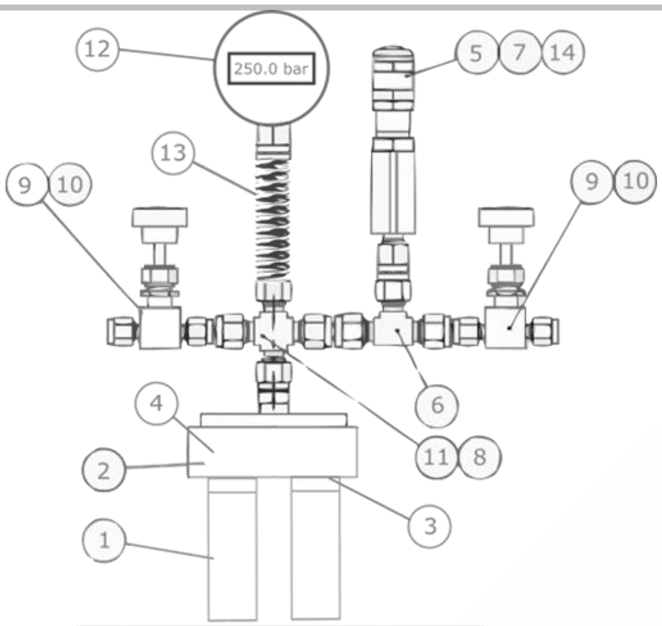

SN	Name	Quantity
1	10 ml Quadracell Reactor	4
2	Quadracell Cap	1
3	Reactor O-Ring-FFKM	4
4	Head closure O-Ring-FFKM	1
5	Presure Relief Valve	1
6	TTE, Equal	1
7	Port Connector	2
8	Tube Cross	1
9	Needle Valve	2
10	Reducing Port Connector	2
11	Adaptor	1
12	Digital Pressure Gauge, 0-250 bar	1
13	Cooling tower	1
14	Spring Kit for PRV 172.3 -241.4 bar	1

Figure S1. Asynt Quadracell reactor setup, featuring an integrated cooling tower and digital pressure gauge.

The overall methodology for both the suspension-based and conventional Kolbe-Schmitt reactions, up to the formation of salicylic acid, is depicted in **Figure S2**. In the suspension-based process, toluene (indicated by an asterisk) was added before the reaction to form a suspension with sodium phenoxide. In contrast, in the conventional process, toluene was introduced only after the reaction to extract the phenolic compounds formed during the reaction.

WILEY ▪ VCH

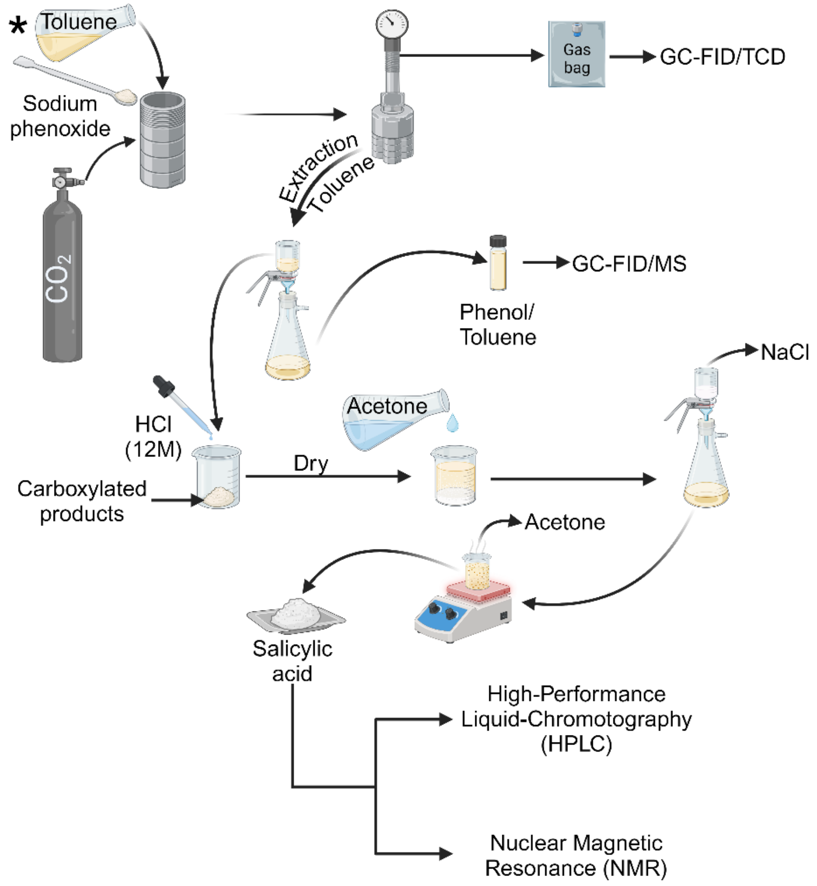

Figure S2. The overall methodology for both the suspension-based and conventional Kolbe-Schmitt reactions leading to the formation of salicylic acid. In the suspension-based reaction, toluene (indicated by an asterisk *) was added before the reaction, while in the conventional process, toluene was introduced after the reaction for extracting the phenolic compounds. (Created in BioRender. Mohammad, O. (2024) BioRender.com/o66g329 [2])

Characterization of gasses by Gas-Chromatography Flame Ionisation Detector and Thermal Conductivity Detector (TCD)

The gas collected in the Tedlar bag was analysed using a Shimadzu GC-2014 gas chromatograph. The analytical conditions for this instrument have been previously reported by the research group[6] . Multiple analyses of the gas samples were performed, and the chromatograms indicated no detection of hydrocarbon gases (**Figure S3**, (a)), with only unreacted CO_2 present (**Figure S3**, (b)).

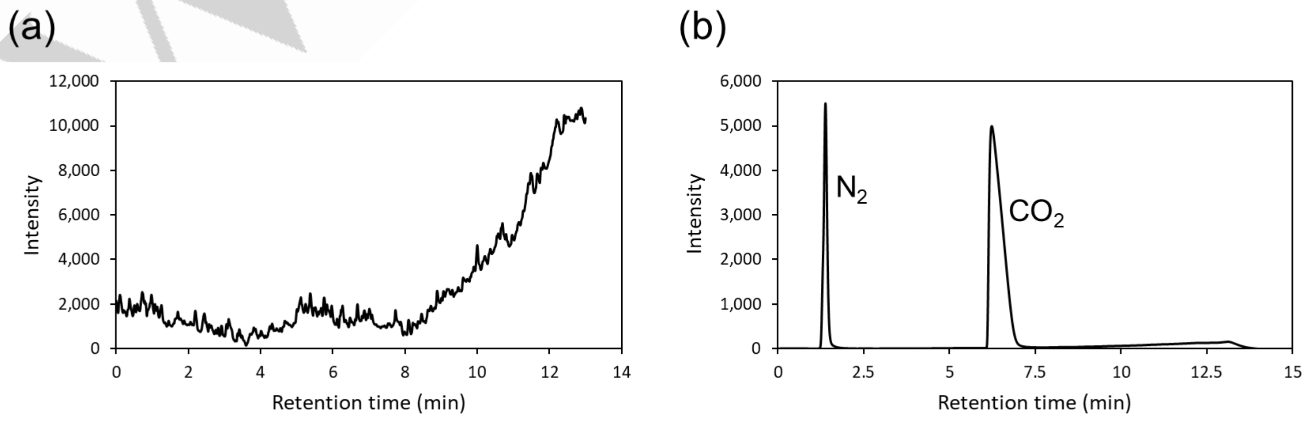

Figure S3. (a) GC-FID analysis showing no detection of hydrocarbons, and (b) GC-TCD analysis indicating the presence of unreacted CO_2 and N_2, which was used as the eluent.

Purity test of the produced sodium phenoxide

The purity of the synthesised sodium phenoxide (PhONa) was evaluated using two methods:

1. **Back-Acidification and NaCl Recovery:**
   PhONa was acidified to yield one mole each of phenol and NaCl (**Scheme S1**). The NaCl was recovered by drying the excess water, followed by acetone treatment to remove any residual phenol. The acetone-containing phenolics were filtered using a Buchner funnel, leaving NaCl crystals on the filter paper. The crystals were dried to remove acetone, and the recovered NaCl mass was compared to the theoretical value (**Table S1**). Results showed nearly 100% NaCl recovery, confirming the high purity of PhONa.

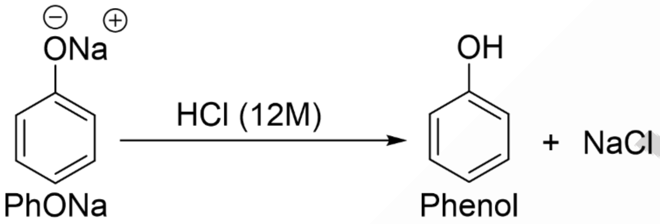

Scheme S1. Acidification of sodium phenoxide (PhONa) to yield one mole of phenol and one mole of NaCl.

Table S1. Results for NaCl recovery post-acidification of sodium phenoxide (PhONa)

**Molecular Weight (g/mol)**	**Phenolic Salt**	**Initial Mass (g)**	**Experimental NaCl (g)**	**Theoretical NaCl (g)**	**Recovered (Purity) (%)**
116.11	PhONa	1.0449	0.5248	0.5259	100%

2. **Acidification and HPLC analysis.**

A known mass of PhONa was acidified after being taken out of the vacuum oven in a 10 mL vial using droplets of concentrated HCl (12M), and the remaining volume was filled with a water-acetone solution (50% v/v). This ensured complete dissolution of phenol and NaCl. The resulting solution was analysed using High-Performance Liquid Chromatography (HPLC). A calibration curve for phenol in water-acetone (50% v/v) was constructed, yielding an R^2 value of 1 (**Figure S4**). Using the calibration curve, the average phenol recovery was calculated as 97.6 ± 0.70%, where 0.70 represents the standard deviation from three replicates. The missing mass was attributed to moisture or minor impurities. These results confirm that highly pure PhONa was used, ensuring accurate mass balance for subsequent reactions. The detailed HPLC method used is provided in the next section, which is the same as that used for the characterisation of HBAs.

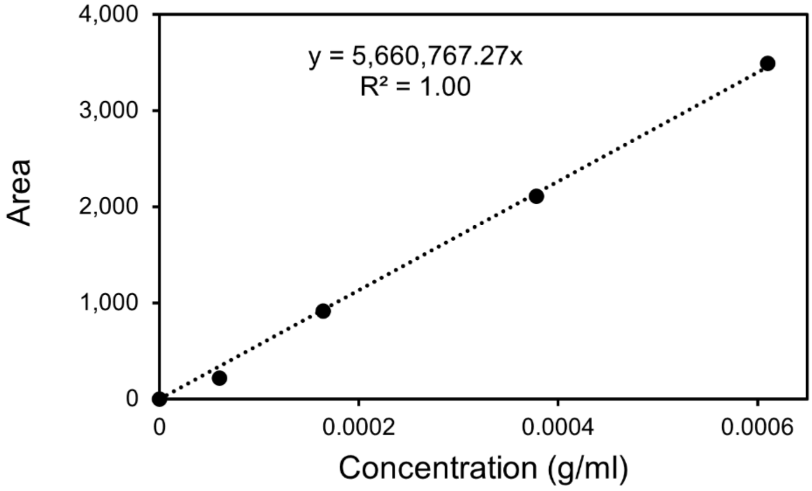

Figure S4. Calibration curve for phenol in a water-acetone (50% v/v) solution used for HPLC analysis.

Characterization of phenol by Gas-Chromatography Flame Ionization Detector and Mass Spectroscopy (GC-FID/MS)

After the reaction, a significant amount of phenol was detected in both the suspension-based and conventional Kolbe-Schmitt reactions. In the suspension-based process, phenol was readily soluble in the toluene used as the dispersion medium. For the conventional process, the reactor and carboxylated products were washed with approximately 50 mL of toluene after the reaction to dissolve the phenol formed during the reaction. A calibration curve for phenol in toluene was constructed, using an external standard method for quantification via GC-FID. GC-MS was employed as a qualitative method to detect any other potential toluene-soluble organics The results confirmed that phenol was the only toluene-soluble organic formed (see **Figure S5**). Full details of the calibration method and the GC-FID/MS analysis are available in a recent publication [1].

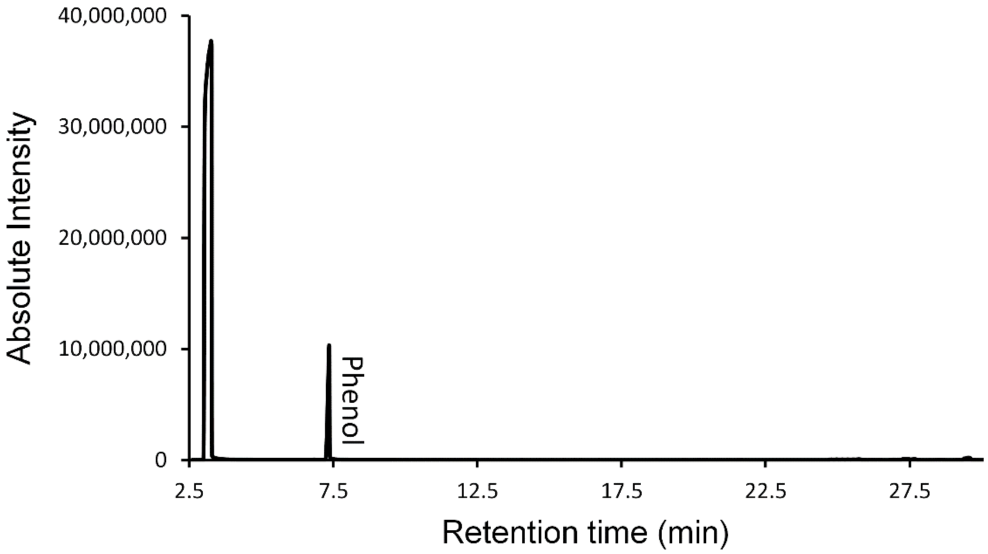

Figure S5. GC-MS spectrum of the toluene-soluble organics. The first peak corresponds to toluene, followed by the only detectable peak of phenol, which was the sole detectable product in the toluene fraction after both the suspension and conventional Kolbe-Schmitt reactions.

For the **suspension-based reaction**, the free phenol yield was calculated as follows:

1. Prior to the reaction, a known mass of phenol was dissolved in toluene to prepare the reaction solution.
2. A 100 µL aliquot of this phenol-toluene solution was taken both before and after the reaction.

3. Each 100 µL aliquot was further diluted in 10 mL of toluene using a calibrated volumetric flask to ensure precise and consistent sample preparation.
4. The concentrations of phenol in these diluted solutions were measured using gas chromatography with flame ionisation detection (GC-FID). The GC-FID analysis relied on a calibration curve of phenol in toluene, which was constructed beforehand to relate signal intensity to phenol concentration.
5. The mass of phenol in the reaction solution before and after the reaction was calculated using the calibration data and the known dilution factors.
6. The difference in mass between these two measurements represents the mass of free phenol formed during the reaction.

For the **conventional Kolbe-Schmitt reaction**, the calculation followed a similar principle but with an initial known mass of phenol added to the reaction:
1. The total initial mass of free phenol was recorded before the reaction commenced.
2. After the reaction, the same GC-FID protocol was used to determine the total free phenol in the reaction mixture. A 100 µL aliquot of the phenol-toluene solution was diluted in 10 mL of toluene, and the phenol concentration was measured using the previously established calibration curve.
3. The difference between the post-reaction phenol mass and the initial phenol mass provided the incremental mass of phenol formed.

Calculations

The molar yields of all hydroxybenzoic acids (HBAs)—salicylic acid, 4-hydroxybenzoic acid, 2-hydroxyisophthalic acid, and 4-hydroxyisopthalic acid—were calculated based on the initial amount of sodium phenoxide (0.3 g) added to each reactor. The simplified stoichiometric equation is shown in **Scheme S2**.

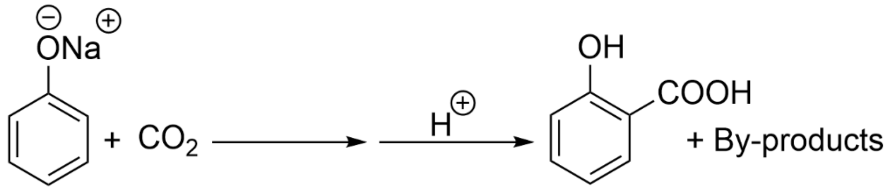

Scheme S2. Simplified stoichiometric reaction equation for both the conventional and suspension-based Kolbe-Schmitt reactions.

Based on this stoichiometric equation, the molar yields of all products were determined using a 1:1 molar ratio, as shown in Equation S1:

$$Yield\ (\%) = \frac{Obtained\ Yield}{Theoretical\ Yield} \times 100 \quad \textbf{(S1)}$$

Additionally, the conversion was calculated as it was possible to quantify all the compounds formed during the reaction. The conversion is expressed by Equation S2:

$$Conversion\ (\%) = \frac{nPhONa_{reacted}}{nPhONa_{fed}} \times 100 = \frac{\sum_n products}{nPhONa_{fed}} \times 100 \quad \textbf{(S2)}$$

.
Where $nPhONa$ represents the moles of sodium phenoxide, and $\sum_n products$ is the total moles of products formed, including the phenols present in the organic phase and all the hydroxybenzoic acids (HBAs) in the solid fraction.

To further validate this method, we ensured that the sum of all product moles $\sum_n products$ and any unreacted sodium phenoxide $nPhONa_{unreacted}$_accounted for the total sodium phenoxide initially fed into the reactor $nPhONa_{fed}$_fed, as in Equation S3.

$$nSA + n4HBA + n2HIPA + nPH_{toluene} + nPhONa_{unreacted} \leq nPhONa_{fed} \quad \textbf{(S3)}$$

Characterization of Hydroxybenzoic acids (HBAs) by High-Performance Liquid-Chromatography

A high-performance liquid chromatography (HPLC) method was developed to effectively separate the products of the Kolbe-Schmitt reaction. A calibration curve of salicylic acid, 4-hydroxybenzoic acid and 2,3-dihydroxyterepthalic acid as a representative for 2-HIPA and 4-HIPA constructed by preparing known masses of the organics dissolved in a water and acetone (50% v/v) solution. For all products, a calibration line of a R-squared value of 1 was achieved. The HPLC method employed a reverse-phase Kinetex 5 µm C18

WILEY - VCH

100 Å, 250 x 4.6 mm column supplied by Phenomenex LTD, UK with a mobile phase consisting of water with 0.1% formic acid (Solvent A) and methanol (Solvent B). The gradient program was as follows: 5.0 min (90% A, 10% B), 20.0 min (70% A, 30% B), 30.0 min (50% A, 50% B), 40.0 min (30% A, 70% B), 45.0 min (10% A, 90% B) and 50.0 min (90% A, 10% B). The flow rate was maintained at 0.5 mL/min with an injection volume of 10 µL, and detection was carried out using a UV detector set at 254 nm with a bandwidth of 4 nm at a column temperature of 30°C. The results of the yield of the hydroxybenzoic acids (HBAs) are in a good accord with the NMR quantification method, hence, provided adequate separation and resolution of the target compound, with consistent retention times and peak areas, demonstrating good reproducibility and suitability for analytical applications.

The total phenols formed during the reaction were recovered and quantified using an external standard method with GC-FID, as detailed in the former section. The fractions of the solid products were determined using HPLC with external standard calibration curves for salicylic acid (SA) and 4-hydroxybenzoic acid (4-HBA). For 2-hydroxyisophthalic acid (2-HIPA) and 4-hydroxyisopthalic acid (4-HIPA), calibration curves of 2,3-dihydroxyterephthalic acid were used as representative standards, since 2-HIPA and 4-HIPA could not be acquired. A mass balance of >95% was achieved for most reactions, confirming the high accuracy of the developed method. The results from GC-FID for phenols and from HPLC for HBAs, for both suspension-based and conventional processes, are summarised in the tables below (**Table S2-S5**).

Table S2. Summary of the results on the effect of reaction time for the conventional Kolbe-Schmitt reaction conducted at 225°C and a CO_2 pressure of 30 bar.

Reaction time	Sodium Phenoxide	Toluene-Soluble-Phenol	Organics	SA	4-HBA	2-HIPA	4-HIPA	Other
h	g	g	g	Product composition wt%				
1	0.30	0.11	0.16	92±1.84	7±0.51	0±0.02	0±0.00	0±0.59
2	0.31	0.08	0.25	87±1.18	8±0.09	2±0.27	0±0.00	3±0.39
4	0.30	0.06	0.26	85±0.16	10±0.05	3±0.01	0±0.00	2±0.06
6	0.30	0.05	0.27	83±2.01	120.65	3±1.20	0±0.00	1±0.97
8	0.30	0.12	0.18	75±1.20	17±0.65	4±0.40	0±0.00	3±0.56

Table S3. Summary of the results on the effect of reaction time for the suspension-based Kolbe-Schmitt reaction conducted at 225°C, CO_2 pressure of 30 bar and 1000 rpm.

Reaction time	Sodium Phenoxide	Toluene-Soluble-Phenol	Organics	SA	4-HBA	2-HIPA	4-HIPA	Other
h	g	g	g	Product composition wt%				
1	0.30	0.12	0.15	89±0.84	1±0.05	3±0.06	1±0.05	7±0.25
2	0.31	0.12	0.17	88±1.20	1±0.05	5±0.04	1±0.06	5±0.34
4	0.30	0.13	0.18	80±0.42	1±0.42	10±0.02	2±0.04	7±0.23
6	0.30	0.12	0.18	80±0.20	1±0.24	11±0.39	2±0.08	5±0.23
8	0.30	0.10	0.22	76±2.11	2±0.06	12±0.12	2±0.02	7±0.58

Table S4. Summary of the results on the effect of phenol-to-phenoxide addition for the conventional Kolbe-Schmitt reaction conducted at 225°C, CO_2 pressure of 30 bar, with a reaction time of 2 hours.

Phenol as promoter	Sodium Phenoxide	Toluene-Soluble-Phenol	Organics	SA	4-HBA	2-HIPA	4-HIPA	Other
wt%	g	g	g	Product composition wt%				
0	0.31	0.08	0.25	87±0.16	8±0.05	2±0.01	0±0.00	3±0.06
0.2	0.30	0.05	0.28	86±2.63	9±0.48	2±0.13	0±0.00	3±0.81
0.5	0.30	0.04	0.29	89±0.57	8±0.30	2±0.08	0±0.00	1±0.24
1.0	0.30	0.02	0.32	94±0.54	5±0.30	1±0.08	0±0.00	0±0.06

Table S5. Summary of the results on the effect of phenol-to-phenoxide addition for the suspension-based Kolbe-Schmitt reaction conducted at 225°C, CO_2 pressure of 30 bar, with a reaction time of 2 hours.

Phenol as promoter	Sodium Phenoxide	Toluene-Soluble-Phenol	Organics	SA	4-HBA	2-HIPA	4-HIPA	Other
wt%	g	g	g	Product composition wt%				
0	0.31	0.12	0.17	88±1.20	1±0.05	5±0.04	1±0.06	5±0.34
0.2	0.30	0.12	0.18	86±0.86	4±0.05	4±0.02	1±0.06	5±0.25
0.5	0.30	0.11	0.20	88±0.75	5±0.42	4±0.04	0±0.04	3±0.31
1.0	0.30	0.11	0.18	91±0.94	3±0.10	3±0.06	1±0.06	2±0.4

Characterisation by nuclear magnetic resonance (NMR) spectroscopy

All NMR measurements were carried out on non-spinning on a 500 MHz Bruker Advance NEO spectrometer, using a 5 mm iProbe equipped with a z-gradient coil producing a maximum gradient of 50.5 G cm^{-1}. Each NMR sample contained *ca*. 0.01 g of the HBA product, with TMS as a reference, in 1 mL DMSO-d_6 solution. All NMR measurements were performed at 298.15 K and used a Oneshot sequence. The use of viscous DMSO-d_6 as a solvent removed any possible effects of convection from the measurements in bulk solution. Ten magnetic field gradient amplitudes, from 6.4 to 25.7 G cm^{-1}, were used and incremented in equal steps of gradient squared. The gradient encoding time for all experiments was 1 ms and all gradients were half-sine in shape. The diffusion delay time, Δ, was set according to the species studied, to obtain *ca*. 80% attenuation of signals. For each gradient amplitude, 64 transients of 16384 complex data points were acquired for a total experimental time of *ca*. 1 hr. DOSY spectra and associated diffusion coefficients were subsequently produced using the DOSY Toolbox software package [3] (see **Figure S6**, below).

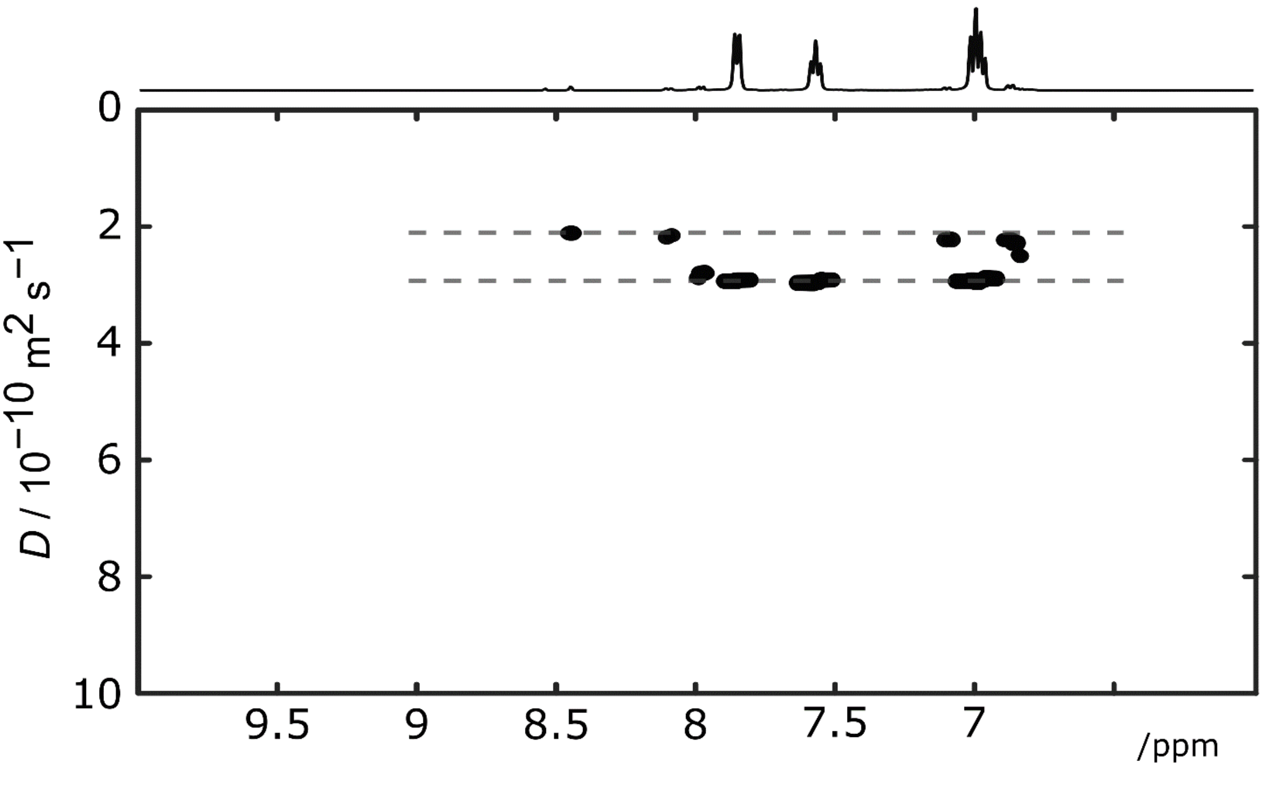

Figure S6. DOSY spectrum of the products of the suspension-based reaction at 2 hours. Horizontal lines aided to indicate species moving at the same speed.

The identification of minor products is facilitated by the DOSY spectrum [4,5]. Horizontal dashed lines highlight proton signals with similar diffusion coefficients. The main product, 2-hydroxybenzoic acid, or salicylic acid, and its isomer, 4-hydroxybenzoic acid, diffuse fastest. The larger, dicarboxylated side products 2-HIPA and 4-HIPA, move more slowly. The two sets of species are thereby separated in the DOSY spectrum. Note that estimation of diffusion coefficients is complicated where peaks overlap, such as around 6.8 ppm in **Figure S6**.

Quantification

Each peak in the spectrum typically represents a single proton, or group of protons, allowing for the assignment of peaks to protons in both major and minor products. By measuring integrals, we can estimate the purity of the product in the sample. **Figure S7** depicts the chemical structures of major products and significant by-products of the reaction.

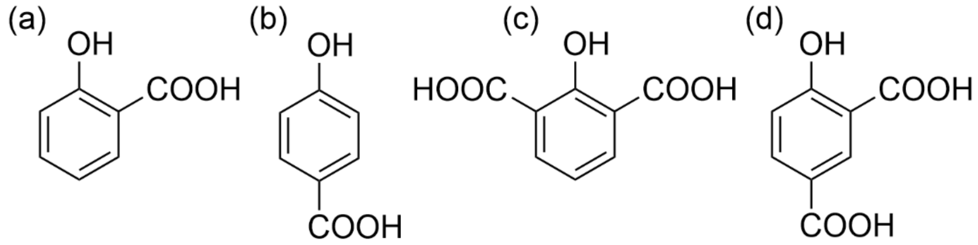

Figure S7. Chemical structures of possible species found in the conventional and suspension-based Kolbe-Schmitt reaction: (a) salicylic acid (SA), the main product; (b) 4-hydroxybenzoic acid (4-HBA); (c) 2-hydroxyisophthalic acid (2-HIPA); and (d) 4-hydroxyisopthalic acid (4-HIPA).

Accurate measurement of integrals relies on identifying isolated peaks in the NMR spectrum. Overlap of peaks belonging to different species will lead to uncertainties in the measurements. The NMR spectrum of the major product, 2-hydroybenzoic acid, consists of two doublets and two triplets. The DOSY spectrum indicates that this species moves fastest of the four isomers identified above in the sample. These four peaks are easily identified. Note the triplet and doublet at lowest chemical shifts overlap with each other. The mono-acid byproduct, 4-hydroxybenzoic acid, exhibits in its NMR spectrum a doublet of doublets, each corresponding to a pair of protons. These can be identified from the DOSY spectrum and coupling constant information as the peaks at 6.8 ppm and 7.8 ppm. The NMR spectrum of the 1,3-diacid spectrum is a doublet with a larger coupling constant, a doublet with a smaller coupling constant and a doublet of doublets. The latter two of these peaks are observed at 8.05 and 8.4 ppm, confirmed by the DOSY spectrum. Finally, the NMR spectrum of the 1,5-diacid consists of a doublet and a triplet. The triplet can be observed at 6.8 ppm. With suitable, isolated peaks identified, **Figure S8** depicts the proton spectra of products of the reaction both when in suspension (top, (a)) and when dry (bottom, (b)), respectively, with integrals, in red, appended for quantification of signals. Concentrations (mol%) based on the normalised integrals are presented in **Table S6**.

WILEY-VCH

WILEY-VCH

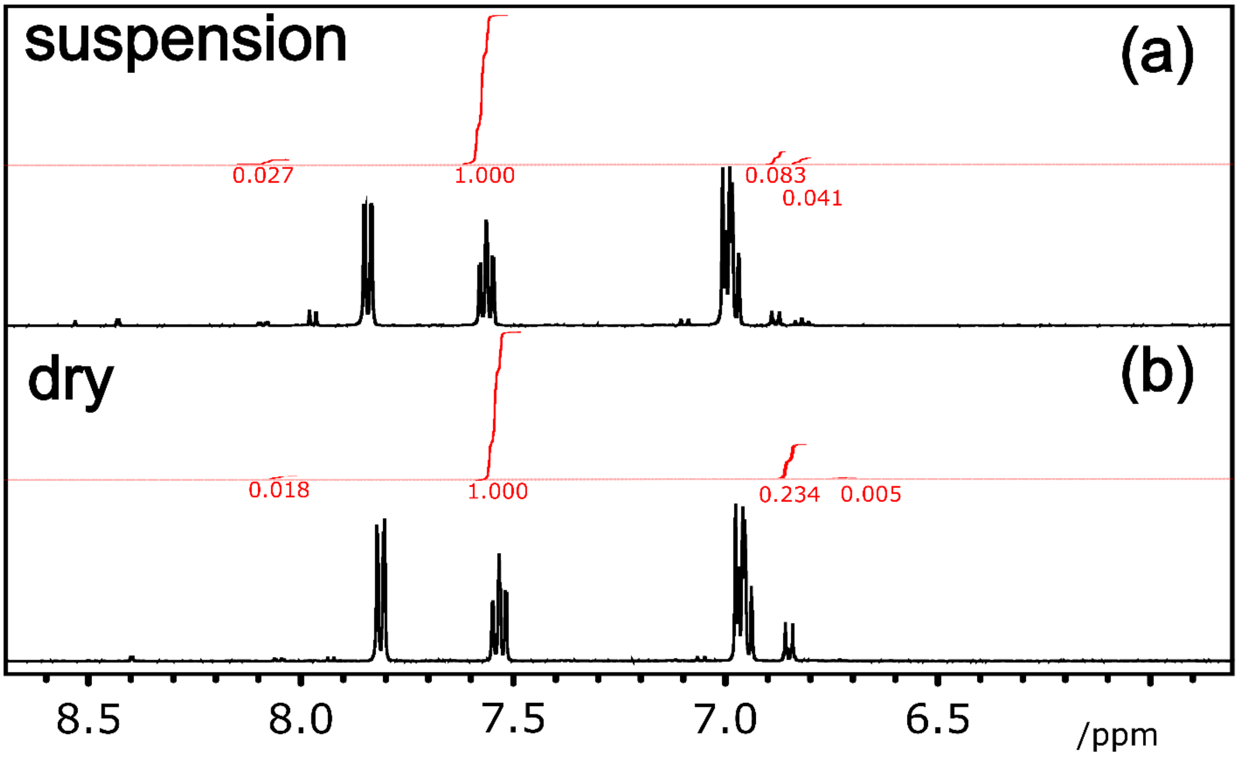

Figure S8. Proton spectra of reaction products of phenol ((a), top) when in suspension and ((b), bottom) when dry. Integrals shown in red and integral data collected in **Table S6**.

The standard deviations between NMR and HPLC for the quantification of Kolbe-Schmitt reaction products, based on concentrations (mol%), are summarised in **Table S6**. These values demonstrate the high accuracy and consistency of the quantification methods used in both HPLC and NMR analyses as the average deviation were <±2%.

Table S6. Average deviations between NMR and HPLC for the quantification of Kolbe-Schmitt reaction products, based on concentrations (mol%)

		Concentration (mol%)			
**Reaction method**	**Analytical method**	o-HBA	p-HBA	2-HIPA	4-HIPA
Suspension	NMR	90.20	3.70	3.70	2.40
	HPLC	94.09	1.08	4.42	0.42
	Average deviation (±)	1.95	1.31	0.36	0.99
Conventional	NMR	87.70	10.30	1.60	0.50
	HPLC	90.20	8.30	1.50	0.00
	Average deviation (±)	1.25	1.00	0.05	0.25

Solubility of CO_2 at various phenol concentrations in toluene

The CO_2 solubility with increasing phenol concentration is based on experimental measurements of the reactor's weight before and after pressurisation to its saturation point (~30 bar) for the suspension-based reaction. The observed mass of CO_2 charged into the reactor, summarised in the **Table S7** below, demonstrates a decreasing trend with higher phenol concentrations.

Table S7. Mass of CO_2 loaded into the reactor based on reactor weight measurements before and after pressurisation (~30 bar) in the suspension-based reaction.

Phenol Concentration	Mass of CO_2 (g)
No Phenol	7.30
20 wt%	7.17
50 wt%	6.83
100 wt%	4.56

Additional references

[1] O. Mohammad, J. A. Onwudili, Q. Yuan, *Molecules* **2024**, *29*, 2527.
[2] Mohammad, O. (2024). The overall methodology for both the suspension-based and conventional Kolbe-Schmitt reactions leading to the formation of salicylic acid. In the suspension-based reaction, toluene (indicated by an asterisk *) was added before the reaction, while in the conventional process, toluene was introduced after the reaction for extracting the phenolic compounds. Created in BioRender. https://BioRender.com/o66g329
[3] M. Nilsson, *J. Magn. Reson.* **2009**, *200*, 296–302.
[4] C. S. Johnson, *Prog. Nucl. Magn. Reson. Spectrosc.* **1999**, *34*, 203–256.
[5] R. Evans, *Prog. Nucl. Magn. Reson. Spectrosc.* **2020**, *117*, 33–69.
[6] C. T. Alves, J. A. Onwudili, *Energies* **2022**, *15*, 7571.

WILEY ▪ VCH
